# “Push–Pull Technique” for the Management of a Selected Superomedial Intraorbital Lesion

**DOI:** 10.1055/s-0038-1661417

**Published:** 2018-06-20

**Authors:** Paolo Castelnuovo, Giacomo Fiacchini, Francesca Romana Fiorini, Iacopo Dallan

**Affiliations:** 1 Division of Otorhinolaryngology, Department of Biotechnology and Life Sciences, University of Insubria-Varese, Ospedale di Circolo e Fondazione Macchi, Varese, Italy; 2 Otorhinolaryngology, Audiology and Phoniatrics Operative Unit, Department of Surgical, Medical, Molecular Pathology and Emergency Medicine, Azienda Ospedaliero Universitaria Pisana, University of Pisa, Pisa, Italy; 3 First ENT Unit, Azienda Ospedaliero-Universitaria Pisana, Department of Surgical, Medical, Molecular Pathology and Emergency Medicine, University of Pisa, Pisa, Italy; 4 Head and Neck Surgery & Forensic Dissection Research Center (HNS&FDRC), Department of Biotechnology and Life Sciences, University of Insubria-Varese, Ospedale di Circolo e Fondazione Macchi, Varese, Italy

**Keywords:** orbital lesion, orbital surgery, intraconal venous malformation, endoscopic transnasal approach, superior eyelid approach

## Abstract

Orbital lesions are traditionally managed through external approaches when laterally located, and through a transnasal approach or other external approaches when medially located. However, when the lesion is superomedially located, it may determine a technical challenge.

In this study, we present the case of a patient with a superomedial intraconal venous malformation of the left eye. We addressed the mass through a combined approach, using the transnasal route as the main approach, and the superior eyelid approach to push down the lesion to facilitate the excision. We have called this approach “push–pull technique.”

We achieved a complete resection of the lesion and did not observe any intraoperative or postoperative complications. The last follow-up at 6 months postoperatively showed no recurrence, and the patient was satisfied and completely recovered.

According to our experience, the “push–pull” technique seems to be a safe procedure and might be considered a valid alternative to address selected superomedial intraconal lesions.

Orbital disease management, although technically demanding and challenging, benefits nowadays several surgical approaches. According to the location, biological behavior, and size of the lesion, the surgeon should design and choose his/her surgical procedure to offer the best angle of attack the lesion itself, thus minimizing the risks to damage noble neurovascular and muscular intraorbital structures.

Laterally located lesions are traditionally managed through lateral orbitotomy or superior eyelid (SE) approach, 1 whereas medially located intraorbital lesions (IOLs) are more easily accessed through the transnasal route or other external transorbital approaches. 2 3 4 5 6 However, certain lesions with superomedial location may determine a technical challenge.

## Materials and Methods

**Fig. 1:**
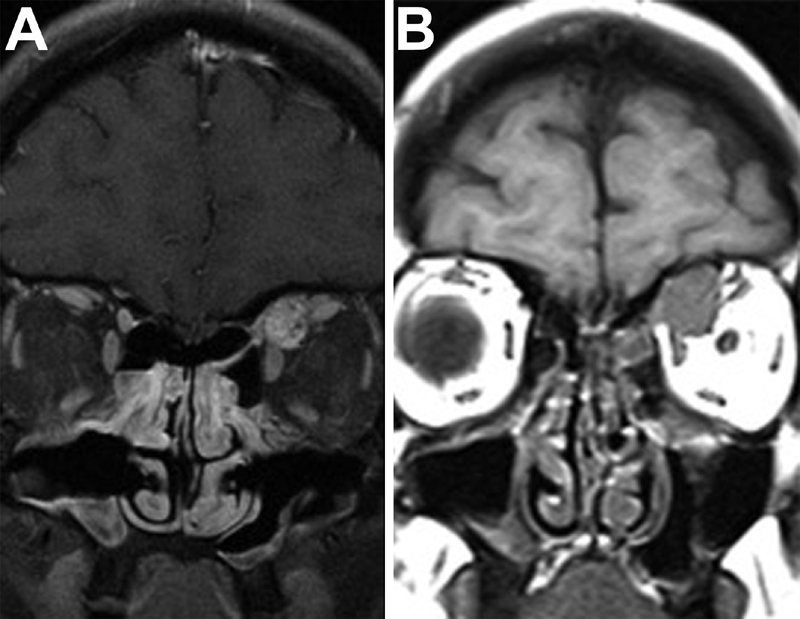
Magnetic resonance imaging (MRI) scan showing a left intraorbital mass increasing in size over the years. ( **A** ) MRI scan taken in 2008. ( **B** ) MRI scan taken in 2016, which shows muscles dislocation.

A 45-year-old woman was referred to the Division of Otorhinolaryngology at the University of Insubria-Varese, Italy, for the incidental finding of a left asymptomatic intraorbital mass, anteriorly located in the superomedial intraconal space, between the superior rectus (SR) and superior oblique (SO) muscles. The imaging suggested a diagnosis of venous malformation. The patient was submitted to a wait-and-scan strategy, and the magnetic resonance imaging (MRI) performed over the years showed a progressive significant increase in the size of the lesion ( Fig. 1 ). In February 2016, the patient started complaining of a left proptosis. The visual field showed left eye central sensitivity reduction, whereas we found left eye visual impairment in the lateral gaze over neurologic examination. After adequate counseling, we obtained a written informed consent to perform the resection of the lesion combining the classical transnasal route and an endoscopic-assisted SE approach. The patient was informed about the possible shift to a traditional neurosurgical external procedure in case of severe intraoperative complications or major difficulties in tumor removal.

**Fig. 2:**
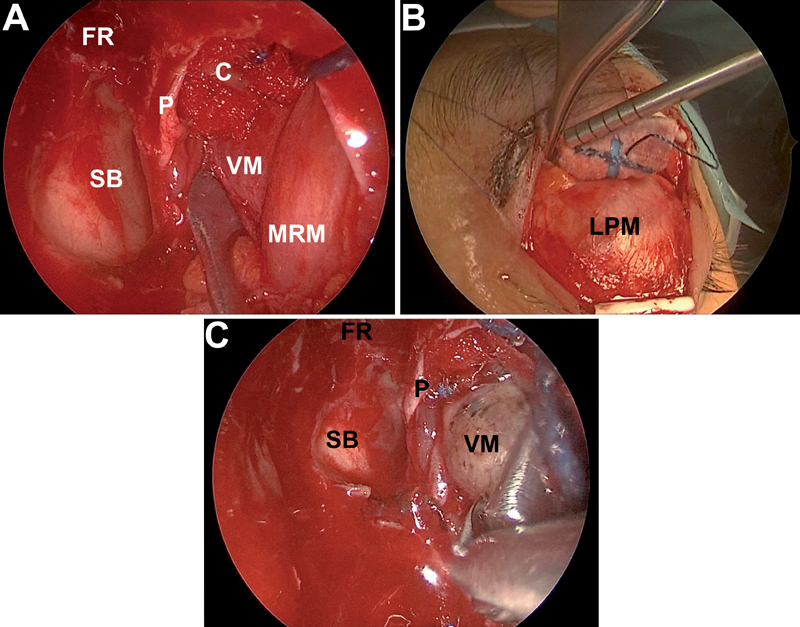
Intraoperative sequential view of the main surgical steps of the “push–pull technique.” ( **A** ) Transnasal exposition of the lesion. ( **B** ) External dissection of the periorbit through a superior eyelid approach. ( **C** ) Endoscopic transnasal view of the lesion with the external push of the periorbit: see the increased exposition of the lesion compared with Fig. 2A . C, cottonoids; FR, frontal recess; LPM, levator palpebrae muscle; MRM, medial rectus muscle; P, periorbit; SB, skull base; VM, venous malformation.

We performed resection of the middle turbinate, complete sphenoethmoidectomy, and opening of the frontal recess. We identified, cauterized, and incised the anterior ethmoidal artery to free the periorbit and mobilize the anterior orbital content. We removed the lamina papyracea and opened the periorbit just in close relationship to the lesion. Once the lesion was visualized, it was dissected free with the aid of cottonoids. Hence, we performed an SE approach. We identified, infiltrated, and incised an adequate palpebral crease. A careful dissection below the orbicularis oculi muscle was performed, and the superolateral border of the orbital rim was reached and skeletonized. After this, a careful subperiosteal dissection of the orbital roof was performed until reaching the medial periorbital region close to the lesion. Having done that, while the lesion was pulled transnasally, from the outside we pushed the orbital content, taking care not to damage the periorbita, in a lateromedial direction to complete the resection of the lesion. In this way, we completed the dissection and resection of the lesion. The periorbit should be spared during the SE approach to make the external push more effective. We have called this approach “push–pull technique” ( Fig. 2 ).

The surgical procedure was performed by a multidisciplinary skull base team with sound experience in extended endoscopic-assisted skull base surgery. The surgical time was approximately 90 minutes. We obtained Institutional Review Board approval for reporting the data.

## Results

**Fig. 3:**
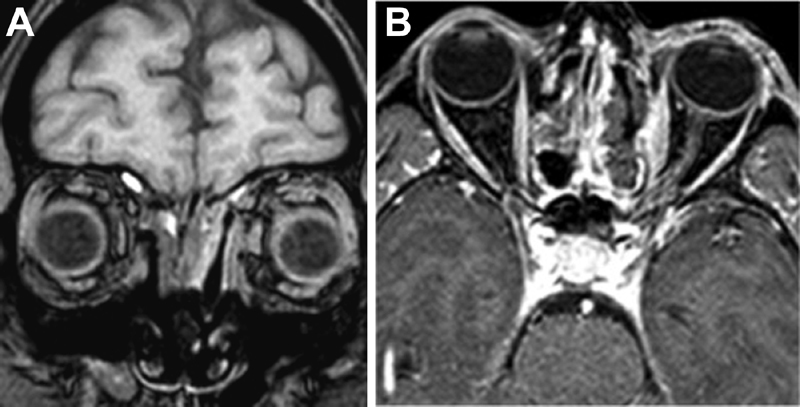
Postoperative magnetic resonance imaging (MRI) scan showing no residual disease. ( **A** ) Coronal view. ( **B** ) Axial view.

We achieved complete resection of the lesion, as confirmed on the postoperative MRI ( Fig. 3 ). We did not observe any intraoperative or postoperative complications. The patient was discharged 3 days after surgery. At the last follow-up, performed at 6 months postoperatively, the patient was satisfied and completely recovered. The palpebral scar was barely visible and was completely hidden with her opened eyes. No residual proptosis or enophthalmos was evident ( Fig. 3B ).

## Discussion

Orbital surgery requires great care to safeguard structural integrity of the vital intraorbital content and associated functions, as well as to preserve the aesthetics of the face. The decision on the surgical approach to undertake should be based on the position, size, and suspected nature of the lesion. In case of well-capsulated intraorbital masses, position and size remain the main criteria to take into consideration when planning surgery.

In this case, we focused on a lesion suspected to be a venous malformation. This is a well-capsulated slowly growing mass, which typically started to cause progressive painless proptosis associated with visual field deficit and eye movements' impairment in a middle-aged woman. The lesion presented an atypical anterior location, in the superomedial intraconal space, between SR and SO muscles.

Recently, the medial orbital space has been addressed through a transnasal corridor. 7 8 If extensive intraconal work is needed, the medial rectus muscle can be medialized and attached to the nasal septum to gain more working room. Furthermore, an anterior septal window can be created to allow a four-hand dissection through the contralateral nasal fossa. Based on our experience, the medial intraconal space may be successfully addressed also through bone-sparing routes such as the transconjunctival one, but in that case, a medial muscle detachment is required and the operative window is quite narrow. As an alternative, according to Leone, 9 this space can be reached by lateralizing the ocular globe after a lateral orbitotomy. This kind of approach, although really interesting, is anyway quite invasive, and, whenever possible, we advise for more conservative procedures.

Recently, the “Pittsburgh group” has proposed an algorithm 10 for the surgical management of orbital disease. The authors compared the orbit to a clock centered on the optic nerve. According to them, the medial transconjunctival approach provides access to the anterior orbit from 1 to 6 o'clock, whereas endoscopic endonasal approaches provide better access to the middle/posterior orbit and orbital apex from 1 to 7 o'clock. We partially agree with their proposal and we found it absolutely logical, although we do not believe that it is not applicable to all types of orbital lesions.

Our case presented a major difficulty, that is, a superomedial and anterior location. Due to the size and the particular position of the lesion, an exclusive transnasal approach would not have been possible due to the different plane of the ethmoidal and orbital roof that obstructs the complete visualization of the lesion. We would like to stress on the anatomical concept that the axial plane passing through the orbital roof is significantly above the axial plane passing through the fovea ethmoidalis, thus making the dissection of the upper part of the lesion (the part above the level of the ethmoidal roof) really complex and uncomfortable. However, we were able to initially dissect the lesion through the nose and complete the dissection after pushing the lesion downward, below the plane of the fovea ethmoidalis.

We believe that the SE approach is a simple and minimally invasive open approach. The incision in the eyelid is quite invisible. As a technical advice, we can underline that a more inferiorly located incision is better from an aesthetic viewpoint given the fact this area is covered when the patient has the eye open. An eyebrow or subbrow incision is by far more evident. This external approach was not needed to visualize and dissect the lesion externally but just to push the lesion medially while pulling it from the nose to complete its resection. The periorbit has to be spared superolaterally, and the orbital content did not need to be entered. No muscle detachment was necessary.

After 6 months, no enophthalmos was evident ( Fig. 3B ). This is probably related to the fact that we opened the periorbita just in close relationship to the lesion, thus reducing unnecessary herniation of the orbital fat and minimizing the disruption of the septa inside of the orbital content. For that reason, we felt that a reconstructive procedure for the medial orbital wall was unnecessary.

Certainly, the lesion could have also been excised through an anterior transpalpebral approach, but we felt more comfortable using a dual-port approach. The final aim of our description is not to propose the “push-pull” technique as the standard of care in such cases but only to present its feasibility in really selected cases and in experienced hands. In term of minimally invasiveness, we would like to stress on the concept that what really matters is not to create damage to noble structures. We feel that the functional aspects represent the most important elements to be considered. In our view, it is not the presence of a skin incision that makes an approach invasive or not. However, we feel that our approach is a well-balanced solution in term of minimal invasiveness, efficacy, and safety. We presented our case just to show that it is possible to combine two different approaches, taking into account the benefits of every approach while minimizing negative aspects.

Finally, from a biological viewpoint, the procedure was safe since the resection could be completed in a one-piece fashion. The patient did not experiment any recurrence since then up to the present day. Last consideration regarding the type of the lesion is that only lesions with expected weak adhesions can be approached through this technique. In lesions with strong adhesions, this kind of technique cannot be proposed. We would like to emphasize the use of cottonoids in this type of dissection. They are very useful in sparing noble structures surrounding the lesion and minimizing surgical trauma. They also allow the surgeon to perform a smooth dissection and keep the surgical field clean, absorbing the blood.

In conclusion, the “push–pull” technique is a safe procedure, which might be considered a valid alternative to address selected superomedial IOLs. However, it should be attempted only by experienced teams able to convert the procedure into a more conventional route and able to face major complications.

Of course, the real applicability of this technique still needs to be confirmed; however, we would like to highlight the importance of modulating the surgical approach according to the needs of the patient.
